# Measuring renal hemodynamics during exercise using doppler ultrasound

**DOI:** 10.14814/phy2.16017

**Published:** 2024-04-16

**Authors:** Christopher L. Chapman, Rachel C. Drew, John R. Halliwill, Christopher T. Minson, Zachary J. Schlader

**Affiliations:** ^1^ Thermal and Mountain Medicine Division U.S. Army Research Institute of Environmental Medicine (USARIEM) Natick Massachusetts USA; ^2^ Military Performance Division USARIEM Natick Massachusetts USA; ^3^ Oak Ridge Institute for Science and Education Oak Ridge Tennessee USA; ^4^ Department of Exercise and Health Sciences University of Massachusetts Boston Boston Massachusetts USA; ^5^ Bowerman Sports Science Center, Department of Human Physiology University of Oregon Eugene Oregon USA; ^6^ Department of Kinesiology Indiana University School of Public Health Bloomington Indiana USA

Dynamic exercise causes a sympathetically mediated reduction in renal blood flow (Rocha et al., 2023). Blood is redistributed away from the renal vascular beds to support the increased metabolic demand of the working skeletal muscle and the skin for thermoregulation (Rocha et al., 2023). This was first demonstrated in 1947 by the seminal work of Barclay et al. (1947), who demonstrated that maximal effort sprinting over 402.3 meters markedly reduced renal plasma flow (diodone clearance) and glomerular filtration rate (inulin or mannitol clearance). Using the reference standard technique of para‐aminohippurate (PAH) clearance, this reduction in renal plasma flow during exercise has since been observed across laboratories using a variety of exercise intensities and environmental conditions (Kenney & Zappe, 1994; Pricher et al., 2004; Radigan & Robinson, 1949).

Our current understanding of the responsiveness of the renal vasculature to the initiation, continuation, and cessation of exercise has been informed in large part by studies using Doppler ultrasonography, a powerful technology that allows the researcher to measure renal hemodynamics with high temporal resolution. A recent review by Rocha et al. (2023) comprehensively described the literature supporting that renal vasoconstriction during exercise is sympathetically mediated and the associated increase in renal vascular resistance and consequent reductions in renal blood flow are evoked by muscle mechanoreflex and metaboreflex stimulation. The cessation of exercise is associated with a rapid decline in the magnitude of renal vasoconstriction from the elevated level occurring during exercise, with previous studies observing that renal vascular resistance returned to resting levels within 40 s at the end of low‐intensity cycling exercise (Endo et al., 2008) and within 5 min following either moderate‐intensity aerobic exercise or high‐intensity anaerobic exercise (Schlader et al., 2019). Notably, Doppler ultrasonography is a challenging technique. Historically, rigorous quantification of volumetric blood flow in the renal artery has been considered unobtainable, as the poor resolution of the vessel walls that lie deep within the human body prohibits the determination of cross‐sectional area (Rocha et al., 2023). However, invasive human studies support the use of renal artery blood velocity as a reliable proxy for renal artery blood flow because pharmacologically induced renal vasoconstriction causes decreases in renal artery blood velocity with no changes in renal artery diameter. Accordingly, vasoconstriction is occurring downstream from these large conduit arteries in the renal afferent and/or efferent arterioles within the kidneys (Rocha et al., 2023).

It is with this background that we read with great interest the recent publication by Kawakami et al. (2024) where it was reported that renal blood flow was maintained during high‐intensity intermittent exercise (HIIE) and moderate‐intensity continuous exercise (MICE), while concluding that “high‐intensity exercise can be performed without a decrease in renal blood flow when performed intermittently for short periods.” In their study, Kawakami et al. employed Doppler ultrasonography to compare the renal hemodynamic responses to HIIE and MICE in 10 middle‐aged male adults. The interpretation of the data presented by Kawakami et al. appears to challenge the well‐established understanding of the renal blood flow response to exercise. However, reconciling these findings with the body of the scientific literature is likely a matter of more carefully considering how methodological choices impacted the quantification of renal hemodynamics.

The authors stated that Doppler ultrasonography was conducted immediately after exercise, but it is unclear how much time elapsed between the cessation of exercise and the visualization of the renal artery, optimization of the imaging, and recording of renal blood flow. According to Figure 1, the HIIE protocol also ended with 2 min of low‐intensity exercise before renal hemodynamics were measured. Given that renal vascular resistance returns to resting levels within 40 s after stopping low‐intensity cycling exercise (Endo et al., 2008), it is plausible that the findings of Kawakami et al. are more reflective of the rapid responsiveness of the renal vasculature to the cessation of low‐intensity exercise than to HIIE. Thus, we believe that the data do not support the conclusion that renal blood flow is maintained during moderate‐intensity and high‐intensity exercise. That noradrenaline, adrenaline, and plasma renin activity were elevated immediately postexercise following HIIE and MICE supports that a sympathetically mediated reduction in renal blood flow occurred during exercise, as has been demonstrated across exercise of various modes, durations, and intensities (Rocha et al., 2023). Rather, Kawakami et al. have captured the immediate postexercise recovery period in which renal hemodynamics are rapidly returned to baseline levels upon the cessation of exercise (Endo et al., 2008; Schlader et al., 2019). This immediate postexercise period is a time of numerous neural adjustments that restore renal blood flow to resting levels, including reduced muscle mechanoreflex and metaboreflex stimulation (Rocha et al., 2023).

We appreciate the challenges associated with employing Doppler ultrasonography during aerobic exercise and have taken measurements after stopping exercise in previous studies (Schlader et al., 2019). However, it is worth noting that this technique has been successfully employed during dynamic exercise (Drew et al., 2013; Endo et al., 2008). It is our collective experience with Doppler ultrasonography that the resolution of the vessel walls of the renal artery is not sufficient for accurate measurement of renal artery diameter (Chapman et al., 2024; Drew et al., 2013; Rocha et al., 2023; Schlader et al., 2019). Therefore, we believe Kawakami et al. could provide a valuable addition to the scientific literature by publishing the methods by which they are able to obtain accurate renal artery diameters, the accuracy and precision of these measurements, and how these measurements were validated, so that other laboratories may employ this technique. At present, we have concern for potential inaccuracies with this approach given that Figure 2 presents a value for renal artery diameter, but the vessel walls of the renal artery cannot be clearly delineated.

In summary, we posit that the findings of Kawakami et al. should be considered a comparison of renal hemodynamics between resting values and the immediate postexercise recovery period, and that the data should not be considered to reflect the response of renal blood flow during exercise. Additionally, we encourage the authors to be more transparent in reporting their methodological approach for measurement of renal artery diameter, as this would benefit the entire scientific community.

The opinions or assertions contained herein are the views of the author(s) and are not to be construed as official or as reflecting the views of the U.S. Army, the Department of Defense, or the U.S. Government.

## FUNDING INFORMATION

None.

## ETHICS STATEMENT

None.
